# Stabilities and novel electronic structures of three carbon nitride bilayers

**DOI:** 10.1038/s41598-018-37100-w

**Published:** 2019-01-31

**Authors:** Wanxing Lin, Shi-Dong Liang, Chunshan He, Wucheng Xie, Haiying He, Quanxiang Mai, Jiesen Li, Dao-Xin Yao

**Affiliations:** 1 State Key Laboratory of Optoelectronic Materials and Technologies, School of Physics, Sun Yat-Sen University, Guangzhou, P. R. China; 2 School of Environment and Chemical Engineering, Foshan University, Foshan, P. R. China; 3 School of Materials Science and Energy Engineering, Foshan University, Foshan, P. R. China

## Abstract

We predict three novel phases of the carbon nitride (CN) bilayer, denoted *α-*C_2_N_2_, *β-*C_2_N_2_ and *γ-*C_4_N_4_, respectively. All of them consist of two CN sheets connected by C-C covalent bonds. The phonon dispersions reveal that all these phases are dynamically stable, because no imaginary frequency is present. The transition pathway between *α-*C_2_N_2_ and *β-*C_2_N_2_ is investigated, which involves bond-breaking and bond-reforming between C and N. This conversion is difficult, since the activation energy barrier is 1.90 eV per unit cell, high enough to prevent the transformation at room temperature. Electronic structure calculations show that all three phases are semiconductors with indirect band gaps of 3.76/5.22 eV, 4.23/5.75 eV and 2.06/3.53 eV, respectively, by PBE/HSE calculation. The *β-*C_2_N_2_ has the widest band gap among the three phases. All three bilayers can become metallic under tensile strain, and the indirect gap of *γ-*C_4_N_4_ can turn into a direct one. *γ-*C_4_N_4_ can become an anisotropic Dirac semimetal under uniaxial tensile strain. Anisotropic Dirac cones with high Fermi velocity of the order of 10^5^ m/s appear under 12% strain. Our results suggest that the three two-dimensional materials have potential applications in electronics, semiconductors, optics and spintronics.

## Introduction

Two-dimensional (2D) materials demonstrate many novel electronic and magnetic properties such as high mobility and optical characteristics^1,2^. The low-buckled honeycomb lattice that consists of silicon and germanium atoms, which are called silicene and germanene, have been predicted^3^, and germanene exhibits the quantum-spin Hall effect^4^ and the materials were synthetized soon after their prediction^5,6^. Plumbene is a normal insulator in its free state, and it can turn into topological insulator by electron doping^7^.

The 2D honeycomb monolayer, consisting of nitrogen atoms denoted nitrogene, has been proposed and its electronic properties have been deeply investigated^8,9^. The electronic properties of nitrogene with vacancy and adsorbed adatoms were also analyzed^10^. Around the same time, another allotrope, octagon-nitrogene, was proposed, and its stability and electronic structure have been systematically studied^11,12^.

The number of 2D materials has been restricted due to the limited number of possible geometric structures, and new compound materials that contain more than one elements are increasing gaining attention. With the rapid development of new low-dimensional materials and devices in experiments and applications, the exploration of new 2D materials is in drastic need. Binary compounds based on two types of atoms may possess new phenomenon compared to their element counterparts^13^, for example, the fullerene-like carbon nitride has been investigated deeply by both experiments^14^ and *first-principles* calculations^15,16^, and the bulk structures formed by carbon and nitrogen atoms C_3_N_4_ have been predicted in recent years. Their electronic and optical properties have been studied by the *first-principles* method^17^. Recently, an interesting ferromagnetic ordering has been found in a prospective spintronic material, B_*x*_C_*y*_N_*z*_ monolayers^18^, as well as the amusing Dirac cone have been found in the organic C_4_N_3_H monolayer theoretically^19^. Some other 2D carbon nitride materials can be used in ‘post-silicon electronics’^20^. However, carbon nitride bilayers with 1:1 stoichiometry have not been proposed or studied until now.

We are trying to explore new 2D materials composed of carbon and nitrogen atoms with novel properties based on density functional theory (DFT). As we know, there are four outermost electrons in the carbon atoms, and five in the nitrogen atoms. In order to satisfy the octect rule, the carbon atoms should form four covalent bonds, while the nitrogen atoms should form three covalent bonds. In this paper, we report a systematic study of the stabilities and electronic structures of the previously unknown phases of 2D binary compounds that have formula C_2_N_2_, or C_4_N_4_, and we name them *α-*C_2_N_2_ and *β-*C_2_N_2_, and *γ-*C_4_N_4_, respectively. Our results indicate that all of the three phases are dynamically stable, and all of them are indirect band gap insulators. Some other similar binary compounds with wide band gaps have been predicted in our other studies^21^.

## Results

### Structure and Stabilities

**Figure 1:**
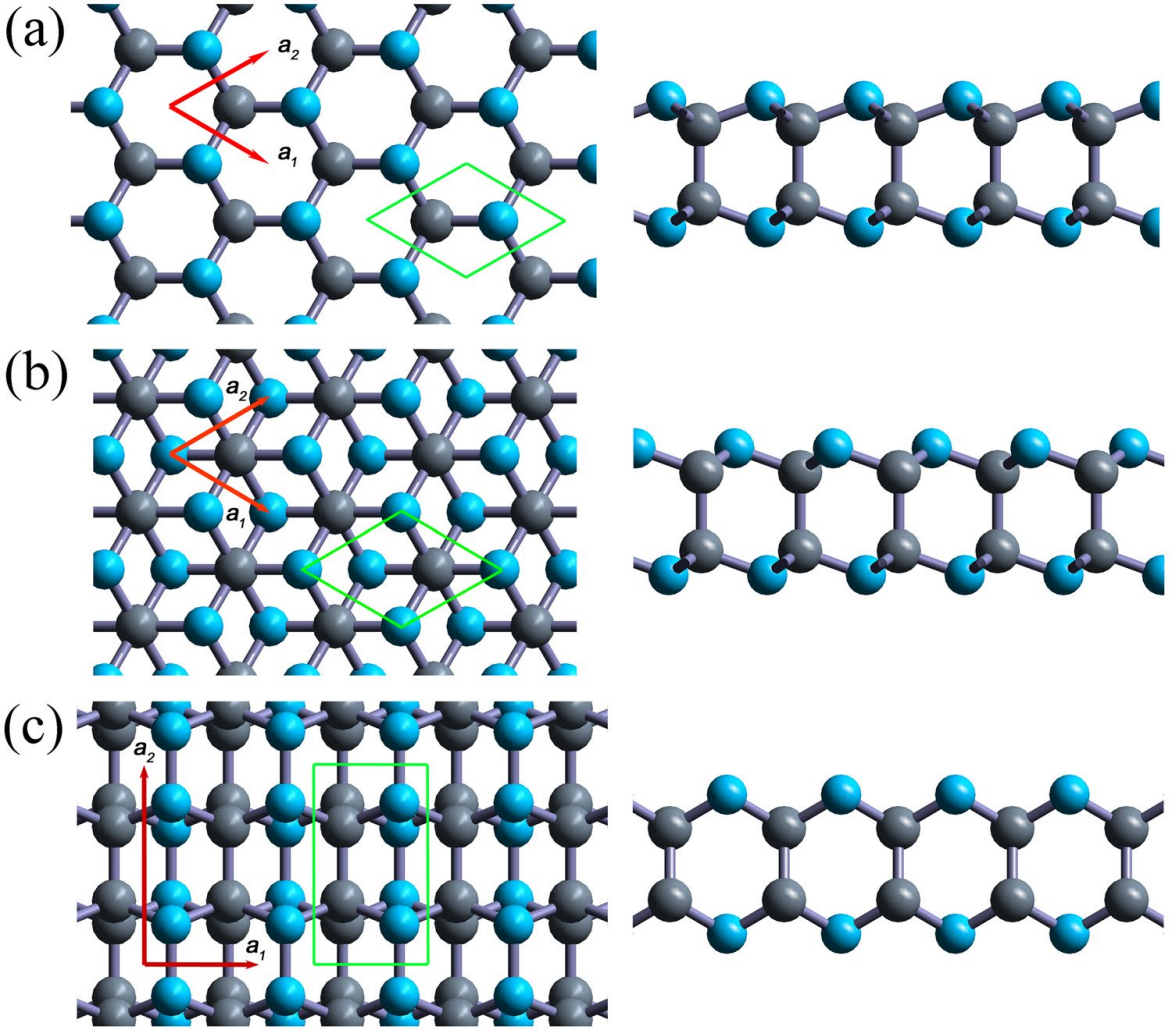
Geometric structures of three carbon nitride bilayers. Top and side views of (**a**) *α-*C_2_N_2_, (**b**) *β-*C_2_N_2_ and (**c**) *γ-*C_4_N_4_. The grey and light blue spheres denote carbon and nitrogen atoms, respectively. Green rhombs are the unit cells, and the red arrows labeled *a*_*1*_ and **a**_*2*_ are the basis vectors.

**Figure 2:**
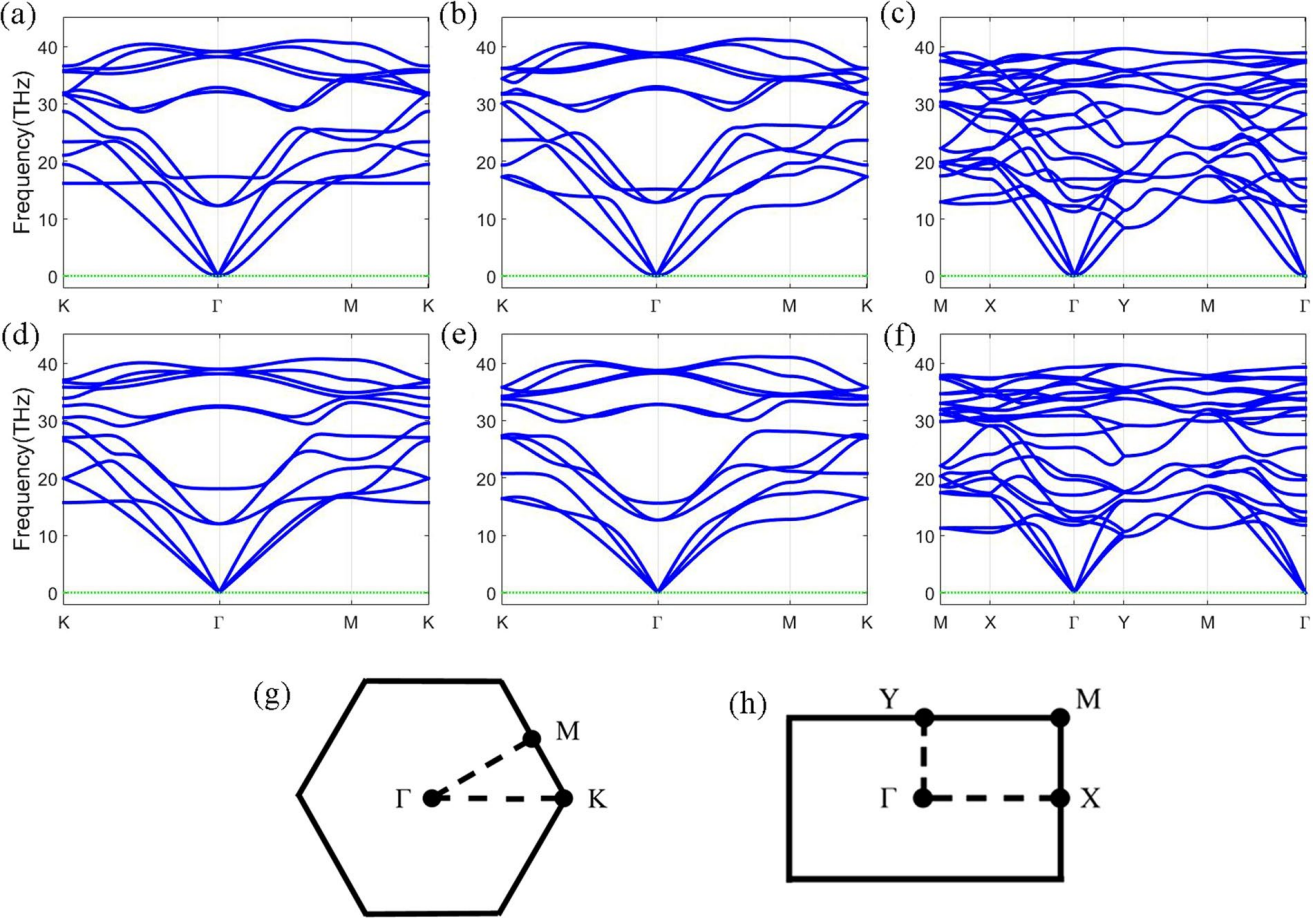
Phonon dispersions of (**a**) *α-*C_2_N_2_, (**b**) *β-*C_2_N_2_, and (**c**) *γ-*C_4_N_4_ bilayers along the HS lines in the BZ calculated by combining VASP with Phonopy, respectively. Phonon dispersions of (**d**) *α-*C_2_N_2_, (**e**) *β-*C_2_N_2_, and (**f**) *γ-*C_4_N_4_ bilayers along the HS lines in the BZ calculated by RESCU. (**g**) is the BZ and the HS points for *α*-C_2_N_2_ and *β*-C_2_N_2_, while (**h**) is for *γ*-C_4_N_4_.

The fully relaxed geometric structures of three carbon nitride bilayers are shown in Fig. 1. The *α-*C_2_N_2_ and *β-*C_2_N_2_ bilayers have stable honeycomb structure, as shown in Fig. 1(a,b). Therefore, the Brillouin zone (BZ) is a regular hexagon, as shown in Fig. 2(g). In Fig. 1(a,b) the basis vectors are denoted by red arrows and the unit cells containing two carbon atoms and two nitrogen atoms, respectively, are denoted as green rhombs. Both of them have the same structure parameters with the in-plane lattice constant 2.35 Å, C-C bond length 1.62 Å, and C-N bond length 1.44 Å. However, they belong to the different point groups: D_3h_ for *α-*C_2_N_2_ and D_3d_ for *β-*C_2_N_2_, respectively. The thickness of bilayer structure is 2.60 Å. The bond angle between the two C-N bonds is 108° 57′, and the bond angle between the C-N bond and C-C bond is 109°58′. Compared with the bond angle of a regular tetrahedral carbon with sp^3^ hybridization, 109°28′, the *α-*C_2_N_2_ and *β-*C_2_N_2_ bilayers show a slight deviation from the standard tetrahedral carbon structure. For the *γ-*phase, we find that *a*_1_ is 2.35 Å, *a*_2_ is 3.99 Å, the N-N bond length is 1.47 Å, the intralayer C-C bond length is 1.54 Å, and the C-N bond length is 1.45 Å, and the thickness of the bilayer is 2.87 Å. The interlayer C-C bond length is 1.55 Å, which corresponds to the interlayer distance. The basis vectors and unit cell are denoted in Fig. 1(c). Its unit cell contains four carbon atoms and four nitrogen atoms. The BZ has a rectangular shape, as shown in Fig. 2(h). Unlike the *α*- and *β*-phases, the *γ*-C_4_N_4_ has D_2h_ symmetry. All three bilayers can satisfy the octect rule.

In order to explore the stabilities of three carbon nitride bilayers, their phonon dispersions have been calculated by both of VASP + Phonopy and RESCU, as shown in Fig. 2(a–c),(d–f), respectively. The absence of vibrational modes with imaginary frequency in the whole BZ, for all of the three phases, suggests that all of them are dynamically stable, and that no frequency gap is found in the dispersions. Moreover, the out-of-plane mode (ZA) around Γ point from RESCU is much stiffer than that from VASP + Phonopy. The wave velocities obtained are listed in Table 1, which shows that both the *α-*C_2_N_2_ and *β-*C_2_N_2_ bilayers are mostly isotropic. However, the *γ*-C_4_N_4_ bilayer exhibits great anisotropy in its mechanical properties, based on their different velocities from the Γ point to the other high symmetry (HS) points.

**Table 1:** Wave velocities of acoustic modes (m/s).

acoustic modes	*α-*C_2_N_2_		*β-*C_2_N_2_		*γ-*C_4_N_4_		
	Γ→M	Γ→K	Γ→M	Γ→K	Γ→X	Γ→Y	Γ→M
ZA	1042 (7214)	1208 (8297)	1222 (8222)	1196 (9299)	607 (3924)	2101 (4729)	2052 (4081)
TA	11554 (8797)	11554 (9782)	11492 (9772)	11498 (11782)	15706 (5095)	11049 (5040)	12631 (6006)
LA	18605 (15245)	18605 (17306)	18411 (14446)	18409 (16066)	18789 (8249)	17759 (8689)	18022 (7577)

The parenthesized numbers are results fitted from RESCU calculations, while the ones that are not parenthesized are calculated by VASP + Phonopy.

**Figure 3:**
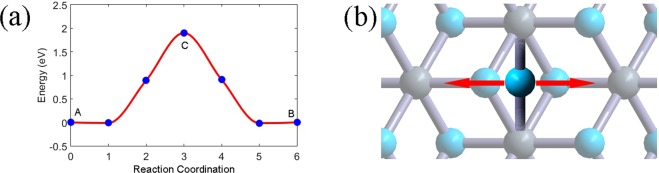
(**a**) Change of total energy of per unit cell during the transformation from *α-*C_2_N_2_ to *β-*C_2_N_2_. The energy of *α-*C_2_N_2_ is set to zero. Blue dots correspond to the inserted images during the CINEB calculation, the point A corresponds to the *α-*C_2_N_2_ bilayer, the point B corresponds to the *β*-C_2_N_2_ bilayer, and the point C corresponds to the transition state. The red curve is the spline interpolation. (**b**) The geometric structure of the transition states. The vibration mode with imaginary frequency is presented by the double-ended arrow, corresponding to the conversion path along the transition.

Due to the similarity between the *α-*C_2_N_2_ and *β-*C_2_N_2_, we have proposed a possible conversion path way between the two phases. From the CINEB calculation, the energy change during the transformation is shown in Fig. 3(a). Since the CINEB can optimize one of the images to the transition state (TS), we study its vibrational mode with imaginary frequency, which corresponds to the conversion pathway, as shown in Fig. 3(b). Obviously, the transition state during the conversion involves the breaking and reforming of C-N bonds, and the transition has a comparatively high energy of 1.90 eV per unit cell with respect to the *α-*C_2_N_2_ and *β-*C_2_N_2_ bilayers, as shown in Fig. 3(a), which is equivalent to an activation energy of 183 kJ/mol, a formidable energy barrier that can prevent reactions under normal conditions.

### Electronic Structures

**Figure 4:**
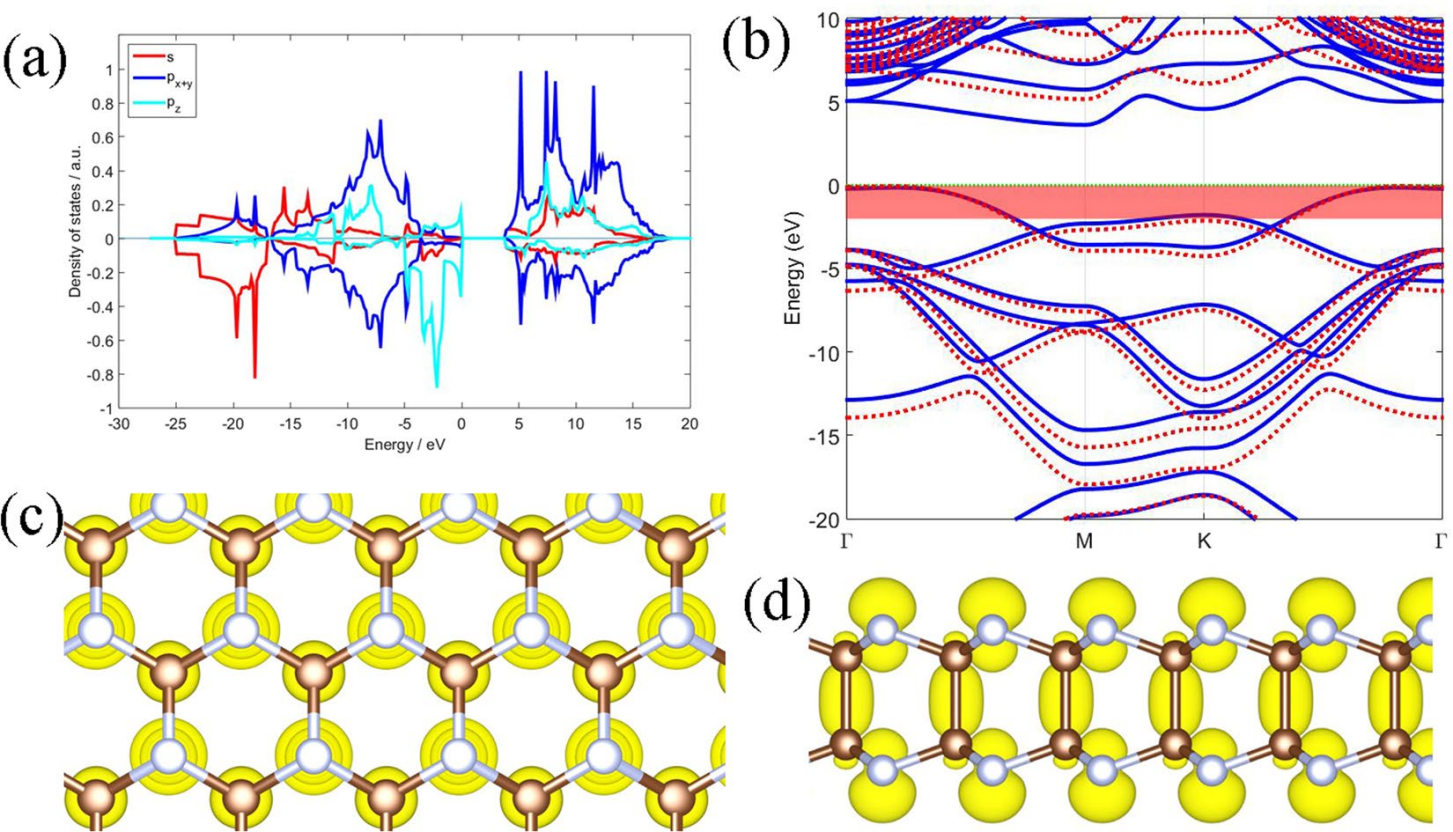
Electronic structures of the *α-*C_2_N_2_ bilayer. (**a**) The density of states: the upper and lower part of the graph represents the C and N components, respectively, and the red, blue and cyan lines represent the s, p_x+y_, p_z_ orbital projected densities of states, respectively. (**b**) The band structures: blue solid/red dotted lines represent the PBE/HSE calculation, the red shaded area indicates −2~0 eV with respect to the Fermi level of the PBE calculation. (**c**) Top and (**d**) side view of the charge density of the states in the shaded area of (**b**). Brown and silvery joints correspond to the carbon and nitrogen atoms, respectively. The Fermi energy is set to zero.

In this section, the electronic structures of the three bilayers will be discussed. In order to interpret the orbital properties of the phases, the projected density of states (PDOS) have been calculated. The PDOS of orbitals of *α-*C_2_N_2_ are shown in Fig. 4(a), with the upper and lower panels being the PDOS of carbon and nitrogen, respectively. As shown in Fig. 4(a), most of the low-energy states come from the s orbits of the nitrogen atoms, while states from valance bands near the Fermi energy come from the p_z_ orbit of the nitrogen atoms. In the conducting bands, the p_x+y_ orbit of both elements make a greater contribution than the s orbits and the p_z_ orbits, and the carbon plays a major role in forming the conducting band. The PDOS of the *β-*C_2_N_2_ and *γ-*C_4_N_4_ are similar.

**Figure 5:**
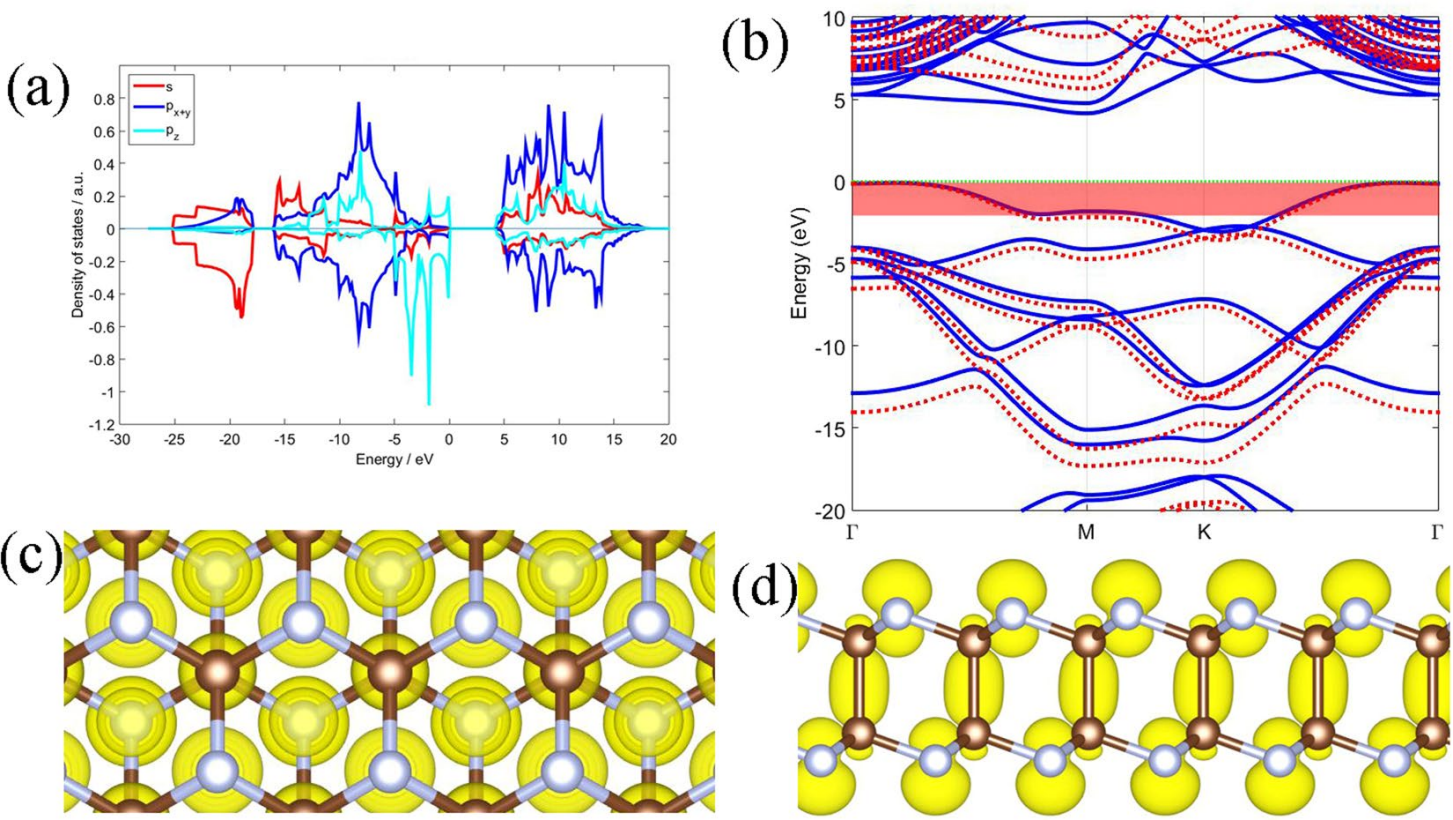
Same as Figure 4 for  *β*-C_2_N_2_ bilayer.

**Figure 6:**
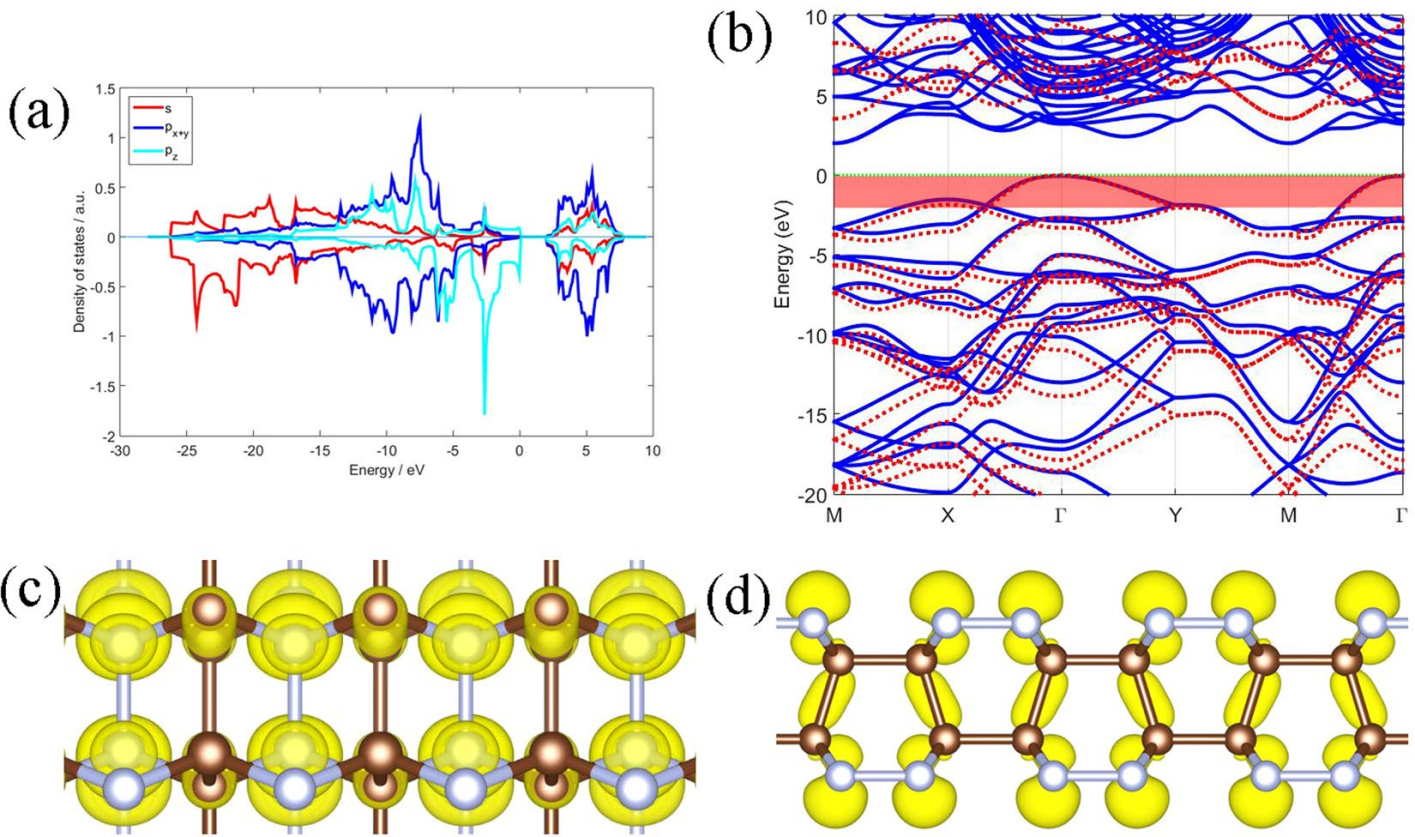
Same as Figure 4 for *γ*-C_4_N_4_ bilayer.

The electronic bands of the *α-*C_2_N_2_ calculated with PBE and HSE functionals are plotted in Fig. 4(b). The results indicate that the *α-*C_2_N_2_ is an indirect band gap semiconductor, with a 3.76 eV band gap by PBE calculation and a 5.22 eV band gap by HSE calculation. The PBE calculation underestimates the band gap by 1.46 eV compared with HSE calculation. The PBE and HSE eigenvalues are nearly ‘degenerate’ near the Fermi level. Figure 5(b) shows that *β-*C_2_N_2_ is also a semiconductor with an indirect band gap of 4.23 eV by PBE calculation and 5.75 eV by HSE calculation. The PBE calculation underestimates the band gap by 1.52 eV compared with HSE calculation. The *γ-*C_4_N_4_ has an indirect band gap of 2.06 eV by PBE calculation, and the HSE calculation gives a wider one with 3.53 eV, which is 1.47 eV wider than the PBE band from Fig. 6(b). The band gap of *α-*C_2_N_2_ is close to the band gap of nitrogene^8,9^, and the *β-*C_2_N_2_ has the widest band gap, while the *γ-*C_4_N_4_ has the narrowest one among them. Furthermore, while the conducting band minimum (CBM) of the three carbon nitride bilayers are all located at the M point, and the valence band maximum (VBM) of the *α-*C_2_N_2_ and *β-*C_2_N_2_ bilayers are located along the K-Γ line, while the VBM of the *γ-*C_4_N_4_ is located at the Γ point.

In order to clarify the bonding mechanism of the structures, the partial charge densities (PCD) are calculated. The charge densities from states from −2~0 eV with respect to the Fermi level of *α-*C_2_N_2_, as indicated in the red shaded area in Fig. 4(b), are shown in Fig. 4(c,d). The charge density distributed around the nitrogen atoms exhibits the character of a p_z_ oribital that points out of the surface. By contrast, the charge density around the C-C bond shows the character of the σ bonds. Most states are located around the nitrogen atoms, which are mostly formed by the p_z_ orbit, because the nitrogen atom possesses one more valance electron than the carbon atom. The other two phases exhibit similar phenomenon, see Figs 5(c,d) and 6(c,d). The nitrogen atoms and carbon atoms in the three structures prefer the sp^3^ hybridization by combining the geometric structures with orbital characteristics.

### Effect of Tensile Strain

**Figure 7:**
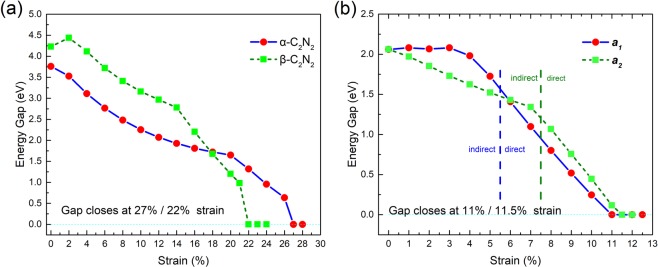
Dependence of band gap on the strains. (**a**) The red dots connected by the blue line and green squares connected by the dark green line represent the dependence of the gap on the biaxial strain of *α-*C_2_N_2_ and *β-*C_2_N_2_, respectively. (**b**) The red dots connected by the blue line and green blocks connected by the dark green line represent the dependence of the gap of *γ-*C_4_N_4_ under strain along ***a***_**1**_ and ***a***_***2***_ directions, respectively.

As previous studies have shown, the electronic structures of nitrogene^9,12^ and phosphorene^22–24^ are sensitive to tensile strain, which means, the band structure can be tuned by strain. In this section, the band structures of the three bilayers have been engineered by tensile strain. All of them undergo an insulator-mental transition under tensile strain. For *α-*C_2_N_2_, as shown by the red dots connected by blue line in Fig. 7(a), the band gap decreases monotonically as the biaxial tensile strain increases, and the band gap closes as the strain reaches 27%. Under the 27% strain, the material turns to two slightly buckled layers with buckling distance 0.007 Å, and the distance between these two layers is 4.446 Å, and the one of nitrogen atoms is 4.453 Å. The bilayer then becomes metallic as the strain continues to increase. For the *β-*C_2_N_2_, the band gap increases until 2%, and decreases monotonically as the biaxial tensile strain increases, and the band gap closes at 22%, as shown by the green dash line in Fig. 7(a). Similar to *α-*C_2_N_2_, the *β-*C_2_N_2_ suddenly becomes two slightly buckled layers under the critical strain of 22%, and the distance between the two layers is 3.67 Å, which is shorter than that for *α-*C_2_N_2_. Because of its different point group and its obvious structural anisotropy, we consider the effect of tensile strain along ***a***_***1***_ and ***a***_***2***_, respectively. Strains along both directions can turn the bilayer into a direct semiconductor and ultimately drive an insulator-metal transition. The band gap decreases almost linearly as the tensile strain increases from 4% to 11% along the ***a***_***1***_ direction, then closes as the strain reaches 11%. Interestingly, the tensile strain along the ***a***_***1***_ direction can turn the bilayer to a direct gap semiconductor with a critical strain of 5.5%, as shown by the red dots connected by the blue line in Fig. 7(b). For the ***a***_***2***_ direction strain, the band gap decreases monotonically as the strain increases and closes at 11.5%. There are two near linear relations from 0% to 7% and from 7% to 11.5%, respectively, and the critical strain of the indirect-direct transition is 7.5%, as shown the green squares connected by the dark green line in Fig. 7(b). The *γ-*C_4_N_4_ bilayer becomes a puckered anisotropic Dirac semimetal as the strains in both directions reach the critical strain.

**Figure 8:**
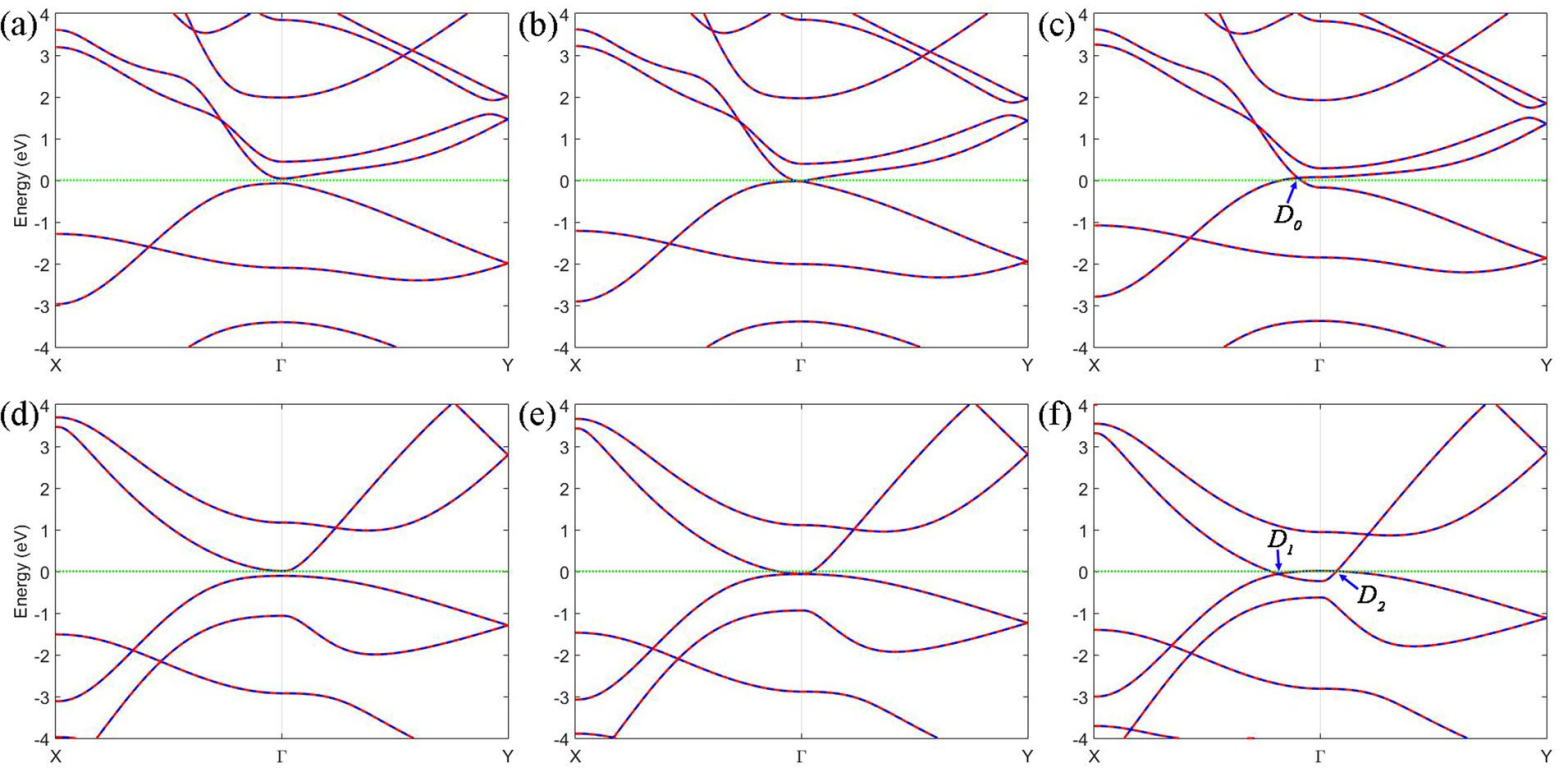
Band structures of the *γ-*C_4_N_4_ bilayer under different strains. Bands of bilayer under strain with (**a**) 10.5%, (**b**) 11%, (**c**) 12% along the *a*_**1**_ direction. Bands of bilayer under strain with (**d**) 11%, (**e**) 11.3%, (**f**) 12% along ***a***_***2***_ direction. The red line and blue dashed line represent the bands without and with considered SOC, respectively. The Fermi energy is set to zero. The Dirac points are denoted as *D*_0_, *D*_1_, and *D*_2_, respectively.

To understand the process of the band gap closing of the *γ-*C_4_N_4_ bilayer under the strains in detail, the band structures around the critical strain have been plotted in Fig. 8. The red line and blue dashed lines represent the band without and with considered SOC, respectively. The Dirac points are denoted as *D*_0_, *D*_1_, and *D*_2_, respectively. Under the strain along the *a*_**1**_, as shown in Fig. 8(a–c), the bilayer becomes a gapless anisotropic Dirac semimetal at the 11% strain, and the CBM and the VBM are degenerate at the Γ point. As the strain along the **a**_**1**_ direction exceeds the critical value, the anisotropic Dirac point shifts away from the Γ point along the Γ-X line. As an example, under 12% strain, a linear dispersion relation appears. The Fermi velocity can be obtained by formula ${v}_{F}=(1/\hslash )\,(\partial E/\partial k)$, where $\hslash$ is the reduced Planck constant. The largest Fermi velocity is 2.69 × 10^5^ m/s at *D*_0_ point as shown Fig. 8(c), while, for the strain along *a*_**2**_, the band gap closes at 11.3% and the CBM and VBM are degenerate at the Γ point. As the strain along *a*_**2**_ increases further, two anisotropic Dirac points appear and move toward to the X and Y points in the BZ, respectively, as shown in Fig. 8(e,f). This is obviously different from the one obtained by stretching along *a*_**1**_. Under the 12% strain along *a*_**2**_, the largest Fermi velocity at the *D*_1_ point is 1.01 × 10^5^ m/s and the largest velocity at the *D*_2_ point is 4.73 × 10^5^ m/s. The energy bands with SOC and without SOC are degenerate in all of the BZ, which indicates the SOC can be neglected in this material. The linear dispersion relation always appears when the strains in the two directions reach some critical values or, in the other words, when the anisotropic Dirac points are robust under the strains.

## Discussion

Three novel phases of carbon nitride bilayers, denoted *α-*C_2_N_2_, *β-*C_2_N_2_ and *γ-*C_4_N_4_, respectively, which satisfy the octect rule, have been predicted by the density functional theory. Their phonon dispersions, transition states and electronic structures have been calculated. Our results show that all of them are stable, while the conversion between the *α-*C_2_N_2_ and *β-*C_2_N_2_ bilayers is found to be hard due to the high energy barrier. The acoustic modes of the phonon dispersions, especially the out-of-plane mode (ZA), appear to be harder than in low-buckled silicene^3^, and black and blue phosphorus^22^. The phonon modes of the three bilayers calculated by the new method RESCU are consistent with the VASP + Phonopy, but harder in the lowest acoustic modes. In terms of energy, both *α-*C_2_N_2_ and *β-*C_2_N_2_ are nearly equally stable. However, the *β* phase is 0.403 meV per atom higher than the *α* phase, and the *γ* phase is 259.59 meV per atom higher than the *α* phase. The formation energies of the proposed phases with respect to graphite and nitrogen gas can be obtained by the formula

1 $${E}_{{\rm{f}}{\rm{o}}{\rm{r}}{\rm{m}}}=({E}_{total}-{n}_{C}\frac{{E}_{graphite}}{4}-{n}_{N}\frac{{E}_{{N}_{2}}}{2})/({n}_{C}+{n}_{N}).$$

Here *E*_graphite_ and ${E}_{{{\rm{N}}}_{2}}$ are the energies of graphite and nitrogen gas, *n*_*C*_ and *n*_*N*_ are the number of C atoms and N atoms in the unit cell, respectively. For *α-*C_2_N_2_ and *β-*C_2_N_2_, *n*_*C*_ = *n*_*N*_ = 2, while for *γ-*C_2_N_2_, *n*_*C*_ = *n*_*N*_ = 4. The formation energies of *α-*C_2_N_2_, *β-*C_2_N_2_, and *γ-*C_4_N_4_ are 409.552 meV/atom, 409.955 meV/atom, and 669.142 meV/atom, respectively. Furthermore, the energy barrier between *α-*C_2_N_2_ and *β-*C_2_N_2_ is 5 meV/atom higher than that from black to blue phosphorus^23^. The electronic bands calculated both by PBE and HSE methods indicate that the three bilayer structures are all indirect semiconductors. Among them, the *β-*C_2_N_2_ bilayer has the widest band gap. Both the phonon dispersions and electronic bands show strong anisotropic properties due to the novel lattice structure of the *γ-*C_4_N_4_ bilayer. The van Hove singularity points from the DOS are similar to graphene^25^, strong electron-electron interactions can be established by applying a strong perpendicular magnetic field. In this case, superconductivity may appear^26,27^. The three bilayers undergo the insulator-metal transitions under strain and the *γ-*C_4_N_4_ bilayer can become a distorted Dirac semimetal with high Fermi velocity under the axial tensile strain. The optimized structures under the strains reveal that the three bilayers are stable as well as flexible, which implies that they may be grown on the suitable substrates. For these bilayers, the critical strains under which the insulator-metal transition take places are greater than those of phosphorene^22,24^. The Fermi velocities under 12% strain in both directions have the same order of magnitudes as the octagons and pentagons graphene-Z^28^, as well as the Dirac monolayer TiB_2_ structure^29^. Obviously, the *γ* phase under the strains is a distorted Dirac semimetal with high Fermi velocity^30^, which possesses more extensive applications than the symmetrical one due to their anisotropic electronic properties at the Dirac point^19^. The *α-*C_2_N_2_ and *β-*C_2_N_2_ bilayers predicted in this work could perhaps be synthesized by the self-condensation scheme from g-C_3_N_4_ materials^31^, or via the wet-chemical reaction as reported for C_2_N-h_2_D^32^. These novel bilayer materials may have promising applications in fields such as semiconductors, spintronics^18^, batteries, supercapacitors^33^ and energy transducers^34^.

## Methods

The optimization and electronic structure calculations have been performed by the Vienna *Ab initio* Simulation Package (VASP) code^35^ within projector augmented wave with cut-off energy of 650 eV, and the exchange-correlation is treated by Perdew-Burke-Ernzerhof (PBE) functional^36^. In addition to PBE calculations, the Heyd-Scuseria-Ernzerhof (HSE06) hybrid functional with a screened Coulomb potential^37,38^ is also used for electronic structure calculation. The energy convergence criterion for electronic iteration in VASP is set to be 10^−8^ eV. The vacuum between two different bilayers is no less than 20 Å. Structures are relaxed until the net force on each ion is less than 10^−4^ eV/Å. The Brillouin zone was sampled with a Γ-centered grid of 20 × 20 × 1 *k* points. Phonon dispersions were calculated using both VASP interface of Phonopy^39^ and a Real space Electronic Structure CalcUlator (RESCU)^40^, respectively, for comparison. In the phonon calculation with VASP + Phonopy, force constants were obtained from a 4 × 4 × 1 supercell for *α*-C_2_N_2_ and *β*-C_2_N_2_, and a 4 × 6 × 1 supercell for *γ*-C_4_N_4_. In the phonon calculation with RESCU, the dimensions of the supercell become 2 × 2 × 1 for *α*-C_2_N_2_, 4 × 4 × 1 for *β*-C_2_N_2_ and *γ*-C_4_N_4_. In RESCU calculation, which is performed in real space grid, double-zeta numerical atomic orbital basis is used to generate Hilbert subspace for the Kohn-Sham DFT solver, with Local Density Approximation (LDA) as the exchange-correlation functional. The grid spacing is no greater than 0.4 Bohr. Self-consistence iteration is performed until the global charge variation is less than 10^−5^ electrons for each valence electron, and the total energy variation per valence electron is less than 10^−6^ Hartree. The conversion path between *α-*C_2_N_2_ and *β-*C_2_N_2_ was calculated by the Climbing Image Nudged Elastic Band method (CINEB)^41–43^, in which five images were inserted in-between.

## References

[1] Novoselov KS (2004). Electric Field Effect in Atomically Thin Carbon Films. Science.

[2] Castellanos-Gomez A (2014). Isolation and characterization of few-layer black phosphorus. 2D Materials.

[3] Cahangirov S, Topsakal M, Aktürk E, Şahin H, Ciraci S (2009). Two- and One-Dimensional Honeycomb Structures of Silicon and Germanium. Physical Review Letters.

[4] Liu CC, Feng W, Yao Y (2011). Quantum Spin Hall Effect in Silicene and Two-Dimensional Germanium. Physical Review Letters.

[5] Feng B (2012). Evidence of Silicene in Honeycomb Structures of Silicon on Ag(111). Nano Letters.

[6] Li L (2014). Buckled Germanene Formation on Pt(111). Advanced Materials.

[7] Yu XL, Huang L, Wu J (2017). From a normal insulator to a topological insulator in plumbene. Physical Review B.

[8] Lee J, Tian WC, Wang WL, Yao DX (2015). Two-Dimensional Pnictogen Honeycomb Lattice: Structure, On- Site Spin-Orbit Coupling and Spin Polarization. Scientific Reports.

[9] Li JS, Wang WL, Yao DX (2016). Band Gap Engineering of Two-Dimensional Nitrogene. Scientific Reports.

[10] Kadioglu Y, Aktürk OÜ, Aktürk E, Ciraci S (2017). Functionalization of Single-Layer Nitrogene by Vacancy, Adatoms, and Molecules. The Journal of Physical Chemical C.

[11] Zhang Y, Lee J, Wang WL, Yao DX (2015). Two-dimensional octagon-structure monolayer of nitrogen group elements and the related nano-structures. Computational Materials Science.

[12] Lin W, Li J, Wang W, Liang SD, Yao DX (2018). Electronic Structure and Band Gap Engineering of Two- Dimensional Octagon-Nitrogene. Scientific Reports.

[13] Nie Y, Rahman M, Wang D (2015). Strain induced topological phase transitions in monolayer honeycomb structures of group-V binary compounds. Scientific Reports.

[14] Sjöström H, Stafström S, Boman M, Sundgren JE (1996). Superhard and Elastic Carbon Nitride Thin Films Having Fullerenelike Microstructure. Physical Review Letters.

[15] Gueorguiev GK, Neidhardt J, Stafström S, Hultman L (2005). First-principles calculations on the role of CN precursors for the formation of fullerene-like carbon nitride. Chemical Physics Letters.

[16] Gueorguiev GK, Neidhardt J, Stafström S, Hultman L (2005). First-principles calculations on the curvature evolution and cross-linkage in carbon nitride. Chemical Physics Letters.

[17] Ruan L, Zhu Y, Qiu L, Lu Y (2013). The First-principles study of basic properties of C_3_N_4_. Journal of Anhui University (Natural Science Edition).

[18] Pan H, Sun Y, Zheng Y, Tang N, Du Y (2016). B_4_CN_3_ and B_3_CN_4_ monolayers as the promising candidates for metal-free spintronic materials. New Journal of Physics.

[19] Pan H (2017). C_4_N_3_H monolayer: A two-dimensional organic Dirac material with high Fermi velocity. Physical Review B.

[20] Kessler FK (2017). Functional carbon nitride materials - design strategies for electrochemical devices. Nature Reviews. Materials.

[21] Li, J. *et al*. Novel Phases of Semi-Conducting Silicon Nitride Bilayer: A First-Principle Study. arXiv: 1707, 02819.

[22] Rodin AS, Carvalho A, Castro Neto AH (2014). Strain-Induced Gap Modification in Black Phosphorus. Physical Review Letters.

[23] Zhu Z, Tománek D (2014). Semiconducting Layered Blue Phosphorus: A Computational Study. Physical Review Letters.

[24] Guan J, Zhu Z, Tománek D (2014). Phase Coexistence and Metal-Insulator Transition in Few-Layer Phosphorene: A Computational Study. Physical Review Letters.

[25] Castro Neto AH, Guinea F, Peres NMR, Novoselov KS, Geim AK (2009). The electronic properties of graphene. Review of Modern Physics.

[26] Cao Y (2018). Correlated insulator behaviours at half-filling in magic-angle graphene superlattices. Nature.

[27] Cao Y (2018). Unconventional superconductivity in magic-angle graphene superlattices. Nature.

[28] Su C, Jiang H, Feng J (2013). Two-dimensional carbon allotrope with strong electronic anisotropy. Physical Review B.

[29] Zhang LZ, Wang ZF, Du SX, Gao HJ, Liu F (2014). Prediction of a Dirac state in monolayer TiB_2_. Physical Review B.

[30] Park CH, Yang L, Son YW, Cohen ML, Louie SG (2008). Anisotropic behaviours of massless Dirac fermions in graphene under periodic potentials. Nature Physics.

[31] Goettmann F, Fischer A, Antonietti M, Thomas A (2006). Chemical Synthesis of Mesoporous Carbon Nitrides Using Hard Templates and Their Use as a Metal-Free Catalyst for Friedel–Crafts Reaction of Benzene. Angewandte Chemie International Edition.

[32] Mahmood J (2015). Nitrogenated holey two-dimensional structures. Nature Communications.

[33] Tan C (2017). Recent Advances in Ultrathin Two-Dimensional Nanomaterials. Chemical Reviews.

[34] Wang X (2009). A metal-free polymeric photocatalyst for hydrogen production from water under visible light. Nature Materials.

[35] Kresse G, Furthmüller J (1996). Efficient iterative schemes for ab *initio* total-energy calculations using a planewave basis set. Physical Review B.

[36] Perdew JP, Burke K, Ernzerhof M (1996). Generalized gradient approximation made simple. Physical Review Letters.

[37] Heyd J, Scuseria GE (2003). Hybrid functionals based on a screened Coulomb potential. The Journal of Chemical Physics.

[38] Paier J, Marsman M, Hummer K, Kresse G (2006). Screened hybrid density functionals applied to solids. The Journal of Chemical Physics.

[39] Togoa A, Tanaka I (2015). First principles phonon calculations in materials science. Scripta Materialia.

[40] Michaud-Rioux V, Zhang L, Guo H (2016). RESCU: A real space electronic structure method. Journal of Computational Physics.

[41] Henkelman G, Uberuaga BP, Jónsson H (2000). A climbing image nudged elastic band method for finding saddle points and minimum energy paths. Journal of Chemical Physics.

[42] Henkelman G, Jónsson H (2000). Improved tangent estimate in the nudged elastic band method for finding minimum energy paths and saddle points. Journal of Chemical Physics.

[43] Sheppard D, Terrell R, Henkelman G (2008). Optimization methods for finding minimum energy paths. Journal of Chemical Physics.

